# All Sequential Dip-Coating Processed Perovskite Layers from an Aqueous Lead Precursor for High Efficiency Perovskite Solar Cells

**DOI:** 10.1038/s41598-018-20296-2

**Published:** 2018-02-01

**Authors:** Muhammad Adnan, Jae Kwan Lee

**Affiliations:** 1 Department of Chemistry, Graduate School, Chosun University, Gwangju, 501-759 Republic of Korea; 2 Department of Chemistry Education/Carbon Materials, Chosun University, Gwangju, 501-759 Republic of Korea

## Abstract

A novel, sequential method of dip-coating a ZnO covered mesoporous TiO_2_ electrode was performed using a non-halide lead precursor in an aqueous system to form a nanoscale perovskite film. The introduction of a ZnO interfacial layer induced significant adsorption in the non-halide lead precursor system. An efficient successive solid-state ion exchange and reaction process improved the morphology, crystallinity, and stability of perovskite solar cells. Improved surface coverage was achieved using successive ionic layer adsorption and reaction processes. When all sequential dipping conditions were controlled, a notable power conversion efficiency of 12.41% under standard conditions (AM 1.5, 100 mW·cm^−2^) was achieved for the perovskite solar cells fabricated from an aqueous non-halide lead precursor solution without spin-casting, which is an environmentally benign and low-cost manufacturing processes.

## Introduction

Recently, organic-inorganic lead halide perovskite solar cells (PrSCs) have received significant attention because of their excellent breakthrough power conversion efficiencies (PCEs) of ~21%, making them potential surrogates for conventional silicon-based solar cells^1–10^. Most organometallic halides, particularly alkylammonium lead halides, (RNH_3_) PbX_3_ (R = alkyl, X = Cl, Br, I), are direct-band-gap materials that are primarily used as hybrid organic-inorganic perovskite cores with exceptional electron and hole conduction and photosensitizer performance^11,12^. The coverage, crystallinity, and uniformity of perovskite materials on its substrate are essential for boosting the PCEs of the fabricated devices. Hence, an significant attention has been devoted to the development of efficient fabrication methods for perovskite material layers in PrSCs. A myriad of strategies have been proposed to improve the PCEs with lead sources of PbX_2_, Pb(OAc)_2_, or Pb(NO_3_)_2_^13,14^. Proposed methods include: (1) the sequential deposition from spin-casting of a lead source followed by reaction with an alkylammonium halide, such as methylammonium iodide (CH_3_NH_3_I, MAI), by dipping in solution, spin-casting, or vacuum deposition^2–15^; and (2) the direct spin-casting of a perovskite precursor solution combined with, adduct, thermal annealing, interfacial engineering, solvent-engineering, or processing additive treatment^16–25^. These methods have provided satisfactorily high PCEs; however, the fabricated PrSCs frequently possess small active areas and suffer from substrate size limitation during spin-casting. Thus, it is challenging, yet essential for commercial applications, to develop inexpensive manufacturing processes that facilitate large area perovskite film formation, via efficient routes including dip-coating, doctor-blade methods, and inkjet or roll-to-roll printing^1–26^.

Most studies have used toxic high-polarity aprotic organic solvents, such as dimethylformamide, due to the poor solubility of the lead precursors. Non-halide lead precursors, such as Pb(OAc)_2_ and Pb(NO_3_)_2_, have recently attracted interest because of their compatibility with non-toxic solvents such as water^3^. Heish *et al*. reported a MAPbI_3_ perovskite film fabricated by sequential deposition^27^. An aqueous Pb(NO_3_)_2_ solution was spin-cast onto UV/ozone-pretreated mesoporous TiO_2_ (m-TiO_2_)/compact TiO_2_ (c-TiO_2_) layers, followed by a two-step reaction in the MAI solution. The first step involved PbI_2_ formation via ion-exchange reaction between Pb(NO_3_)_2_ and MAI and the second involved MAPbI_3_ perovskite formation from PbI_2_ and MAI. Although the Pb(NO_3_)_2_ film was uniformly spin-cast onto the m-TiO_2_ layer, the morphology and coverage of the PbI_2_ film formed in the first step was significantly impaired. This impairment was caused by the structural change induced by the difference in ionic radii between I^−^ and NO_3_^−^. In the following step, well-defined morphology and coverage of the MAPbI_3_ perovskite film on the substrate was not achieved compared to those fabricated by spin-cast PbI_2_ films. To overcome these challenges, we have been interested in developing novel and efficient approaches for perovskite film formation. Preparation from aqueous non-halide lead precursor solutions using non-spin-casting methods is a proposed environmentally benign and low-cost manufacturing processes for large area devices. Recently, ionic layer adsorption, followed by reaction of Pb(NO_3_)_2_ on a ZnO heterostructure, in aqueous solution has been reported as an attractive approach for the formation of PbS quantum dot layers^28,29^. Thus, we have attempted to form an efficient MAPbI_3_ perovskite film by the ionic layer adsorption of aqueous Pb(NO_3_)_2_ by introducing a ZnO interfacial layer on top of a m-TiO_2_ film, followed by reaction with MAI. We speculate that this successive sequential deposition (SSD) via dip-coating might facilitate the production of large area perovskite films using non-halide lead precursors in aqueous solution.

In this study, we demonstrate a facile, cost-effective, and environmentally benign approach to prepare efficient perovskite films by simple dip-coating deposition. This was demonstrated by sequentially dipping of a ZnO-covered m-TiO_2_ film in an aqueous lead precursor solution and then the MAI solution. This process contrasts with conventional spin-casting approaches that require harsh organic solvents.

**Figure 1:**
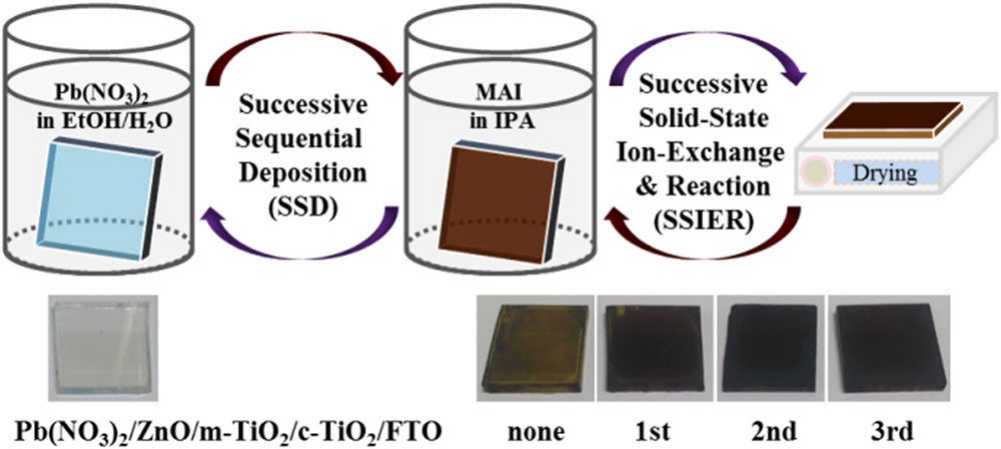
Schematic description of the preparation of MAPbI_3_ perovskite films using sequential dipping of ZnO covered m-TiO_2_/c-TiO_2_/FTO substrates in aqueous Pb(NO_3_)_2_ and MAI solutions. Included are pictures of the as-prepared electrodes after a designated number of SSIER repetitions followed by annealing for 10 min at room temperature.

Herein, we found that the ZnO interfacial layer quickly induces significant adsorption of Pb(NO_3_)_2_ from the aqueous solution. The MAPbI_3_ perovskite was also formed via Pb(NO_3_)_2_ and PbI_2_ undergoes additional ion-exchange reactions with the un-reacted Pb(NO_3_)_2_ even in the solid state, resulting in decomposition to PbI_2_. Notably, the successive solid-state ion-exchange and reaction (SSIER) with the Pb(NO_3_)_2_ layer resulted in improved crystallinity, morphology, and coverage as well as stability of the MAPbI_3_ film compared to materials produced by long-time dipping in MAI solution. Moreover, the perovskite film fabricated with the combined SSD and SSIER techniques exhibited superior crystallinity, morphology, and coverage compared to those made with either approach alone. The PrSCs fabricated by the sequential deposition of a MAPbI_3_ perovskite layer using an aqueous Pb(NO_3_)_2_ solution exhibited PCEs of 12.41%, which is comparable to films fabricated by spin-casting of halide or non-halide lead precursors. Figure 1 shows a schematic description of the preparation of the MAPbI_3_ perovskite films by sequentially dipping a ZnO-covered m-TiO_2_/c-TiO_2_/fluorine-doped tin oxide (FTO) substrate in aqueous Pb(NO_3_)_2_ and MAI solutions.

## Results and Discussion

### Perovskite film formation

**Figure 2:**
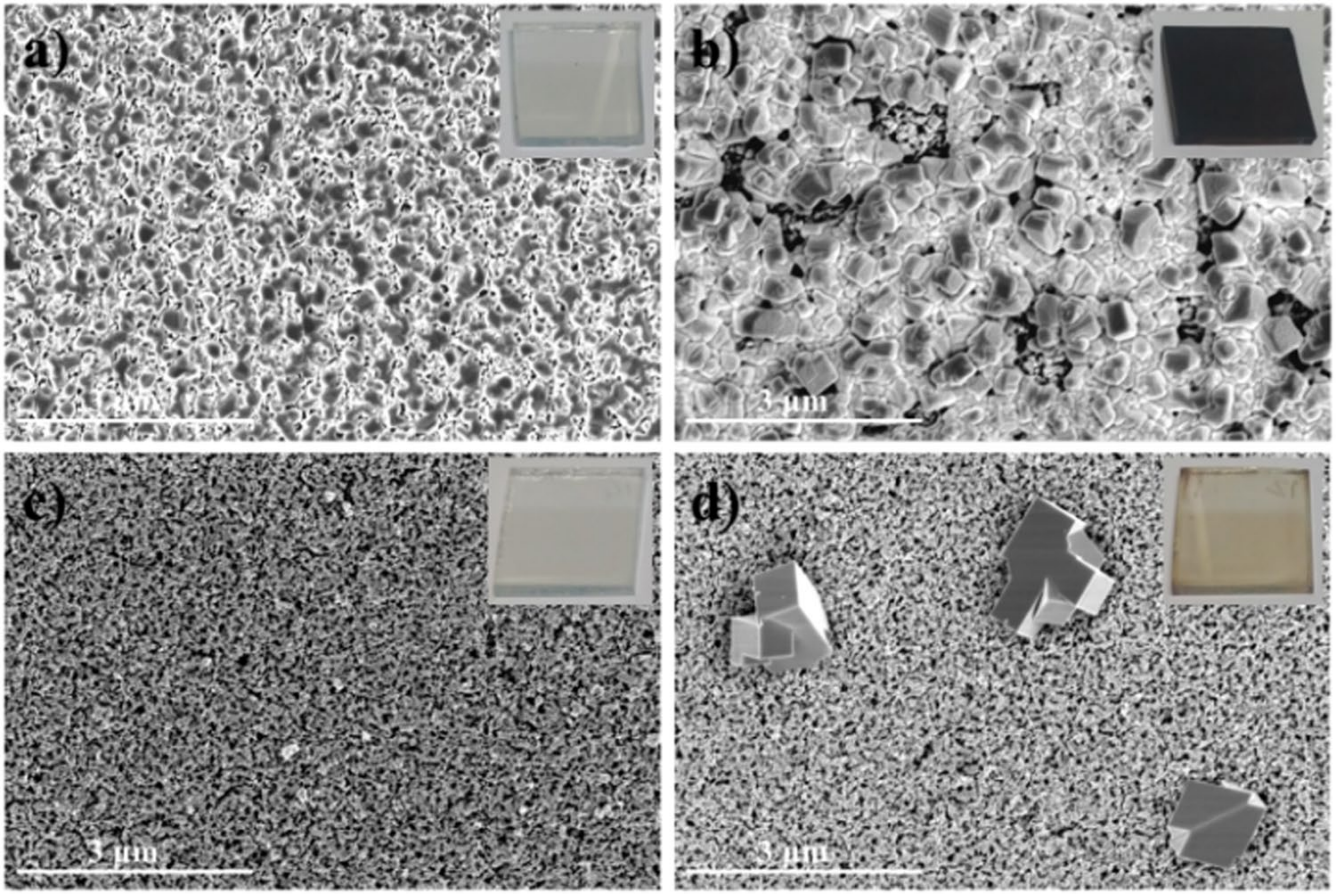
SEM images for surface morphologies from (**a**,**c**) the ionic layer adsorption of Pb(NO_3_)_2_ and (**b**,**d**) the as-prepared MAPbI_3_ perovskite layers formed after the MAI exposure of the (**a**,**b**) ZnO-coated and (**c**,**d**) ZnO-free m-TiO_2_/c-TiO_2_/FTO substrates.

For the formation of perovskites, we first fabricated the Pb layer by dipping the substrate into a Pb(NO3)_2_ solution. Figure 2(a,c) shows the SEM surface morphologies after ionic layer adsorption of Pb(NO_3_)_2_ on m-TiO_2_/c-TiO_2_/FTO substrates with and without a ZnO layer. In Fig. 2(b,d) the as-prepared MAPbI_3_ perovskite layers formed when the ZnO-free and ZnO-coated samples were exposed to the MAI solution can be seen. We chose Pb(NO_3_)_2_ as the non-halide lead precursor because it exhibited better ionic layer adsorption on ZnO surfaces in aqueous solutions (see Fig. S1 in the Supplementary Information). Thin ZnO layer-incorporated m-TiO_2_ films were used because they could be readily prepared by spin-casting or spray-coating of the sol-gel precursor solution, followed by annealing at 300 °C (Fig. S2). Interestingly, significant layer adsorption of Pb(NO_3_)_2_ was observed on the surface of the ZnO-coated substrate after the simple dip-coating process in the aqueous Pb(NO_3_)_2_ solution, but negligible layer adsorption on the m-TiO_2_ films was observed without the ZnO layer (Fig. 2(a) and (b)).

This unprecedented Pb(NO_3_)_2_ layer adsorption occurred on the ZnO surface within a very short time, which can be tuned by adjusting the lead precursor solution, especially the co-solvent system of H_2_O/ethanol. A more uniform adsorption was observed on the hydrophobic ZnO surface compared with the water-only solvent^30,31^. By adsorption can also be changed by tuning the dipping times, as shown in Fig. S3. For MAPbI_3_ perovskite formation from the reaction with MAI, the Pb(NO_3_)_2_ layer was first deposited by dip-coating in a 0.1 M Pb(NO_3_)_2_ solution, dissolved in H_2_O/ethanol (1:1, v/v), for 30 s. As shown in Fig. 1c and d, the transparent Pb(NO_3_)_2_ layers adsorbed on the ZnO layer-coated m-TiO_2_ film. These layers rapidly changed to dark brown-colored films upon exposure to the MAI solution, exhibiting a MAPbI_3_ perovskite film morphology and poor coverage similar to those formed by spin-casting of the MAPbI_3_ precursor solution without any treatment. However, the transparent Pb(NO_3_)_2_ layers, which adsorbed minimally on the pristine ZnO-free m-TiO_2_ film, changed to pale orange-colored films with MAPbI_3_ perovskite crystal lumps grown sparsely on the substrate.

These results indicate that the sequential approach based on the layer adsorption on ZnO of aqueous Pb(NO_3_)_2_ followed by reaction in a MAI solution facilitates perovskite film formation using inexpensive and simple dip-coating deposition processes. However, the MAPbI_3_ perovskite film fabricated by this approach rapidly decomposed during solvent drying at 80 °C, even below 20% relative humidity, the change to a yellowish film corresponded to a PbI_2_ crystalline morphology.

### Decomposition behaviour of perovskite film

**Figure 3:**
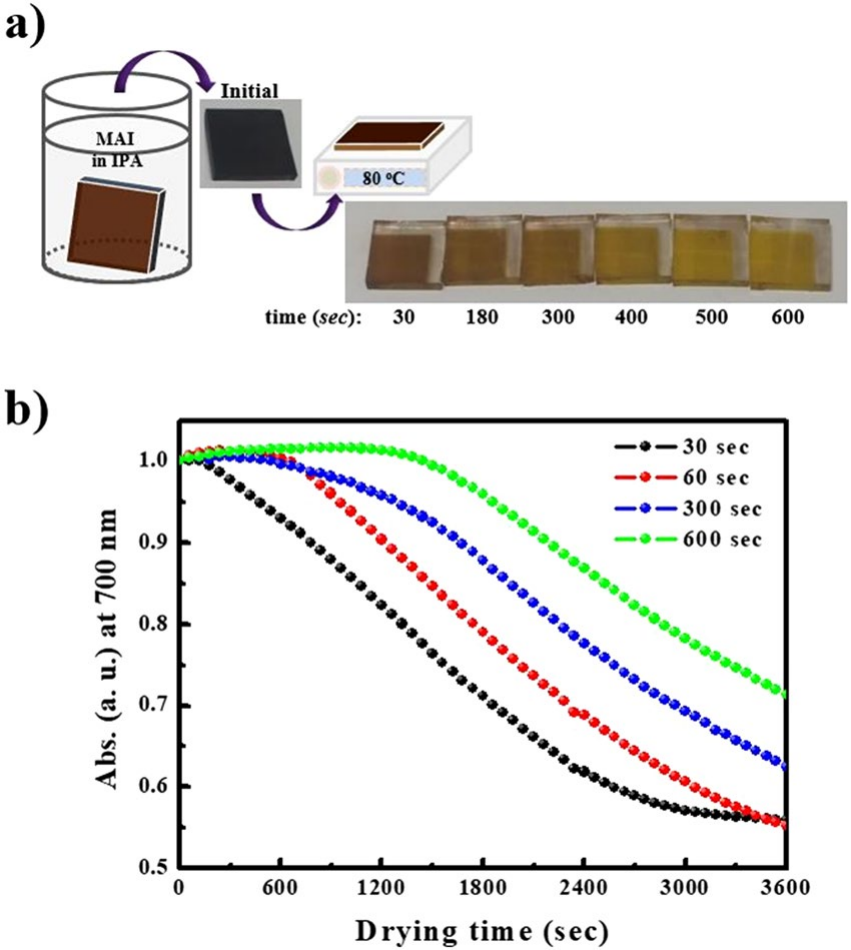
Decomposition (**a**) during solvent drying of a MAPbI_3_ perovskite film, fabricated by sequential dip-coating deposition with aqueous Pb(NO_3_)_2_ precursor and MAI solutions. The decrease of absorbance (**b**) at 700 nm over 1 h after various MAI solution dipping times (30 (black), 60 (red), 300 (blue), and 600 s (green)).

Figure 3 shows the decomposition observed during solvent drying of the MAPbI_3_ perovskite film fabricated by sequential dip-coating deposition. Also shown in Fig. 3b is the decrease of absorbance at 700 nm over 1 h for the MAPbI_3_ perovskite materials formed with various MAI solution dipping times (30, 60, 300, and 600 s). As shown in Fig. 3a, film decomposition occurred within a very short time of 30 s and was completed after 10 min of annealing at 80 °C. Decomposition was partially observed even in films fabricated with a long exposure (600 s) to the MAI solution (Fig. 1).

Thus, we investigated the decomposition rate of the films formed with various dipping times by following the decrease in absorbance at 700 nm. Although the as-prepared MAPbI_3_ perovskite films had a similar dark brown-color upon removal from the MAI solution regardless of the reaction times, the decomposition rates were remarkably affected by the reaction time in the MAI solution (Fig. 3b). This indicates that unreacted Pb(NO_3_)_2_ likely induces the decomposition of the MAPbI_3_ perovskite crystalline layer, and the degree of unreacted Pb(NO_3_)_2_ should affect the decomposition rates. From these results, we speculate that the MAPbI_3_ formed from the Pb(NO_3_)_2_ and MAI solutions can participate in ion-exchange reactions between the unreacted Pb(NO_3_)_2_, even in the solid state, resulting in decomposition to PbI_2_. Therefore, we developed an efficient approach, denoted as successive solid-state ion-exchange and reaction (SSIER). This method prevents the decomposition of the MAPbI_3_ perovskite into PbI_2_ by the ion-exchange reaction in the solid state between unreacted Pb(NO_3_)_2_ and the as-formed MAPbI_3_.

**Figure 4:**
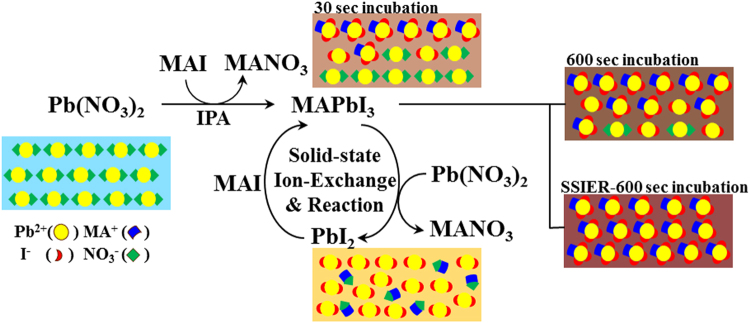
Schematic description of the proposed SSIER approach to MAPbI_3_ perovskite formation from the as-deposited Pb(NO_3_)_2_ layer and MAI.

Figure 4 shows a schematic description of the proposed mechanism of SSIER for MAPbI_3_ perovskite formation. Initially, the PbI_2_ produced from the reaction of Pb(NO_3_)_2_ and MAI at the solid-liquid interface is converted rapidly into MAPbI_3_, which undergoes further reactions with MAI. However, the ongoing diffusion of MAI into the film is obstructed by the bulky MAPbI_3_ perovskite structures formed on the film surface, retarding the reaction rate of Pb(NO_3_)_2_ and MAI. Thus, although the Pb(NO_3_)_2_ layer is exposed to the MAI solution for a long time, unreacted Pb(NO_3_)_2_ likely remains and can induce the decomposition of the MAPbI_3_ perovskite structure, even in the solid state. Meanwhile, the Pb(NO_3_)_2_ inside the film would be removed, as shown in Fig. 4.

We attempted to efficiently achieve more stable MAPbI_3_ films without unreacted Pb(NO_3_)_2_ by applying the SSIER process with a exposure time of 30 s to the MAI solution followed by annealing at 80 °C. This represented a single SSIER cycle. No apparent decomposition of the MAPbI_3_ film was observed after the 3rd SSIER cycle after a total MAI exposure time of 90 s. The MAPbI_3_ film morphologies formed with and without SSIER repetition were also compared.

### Characterization of perovskite film

**Figure 5:**
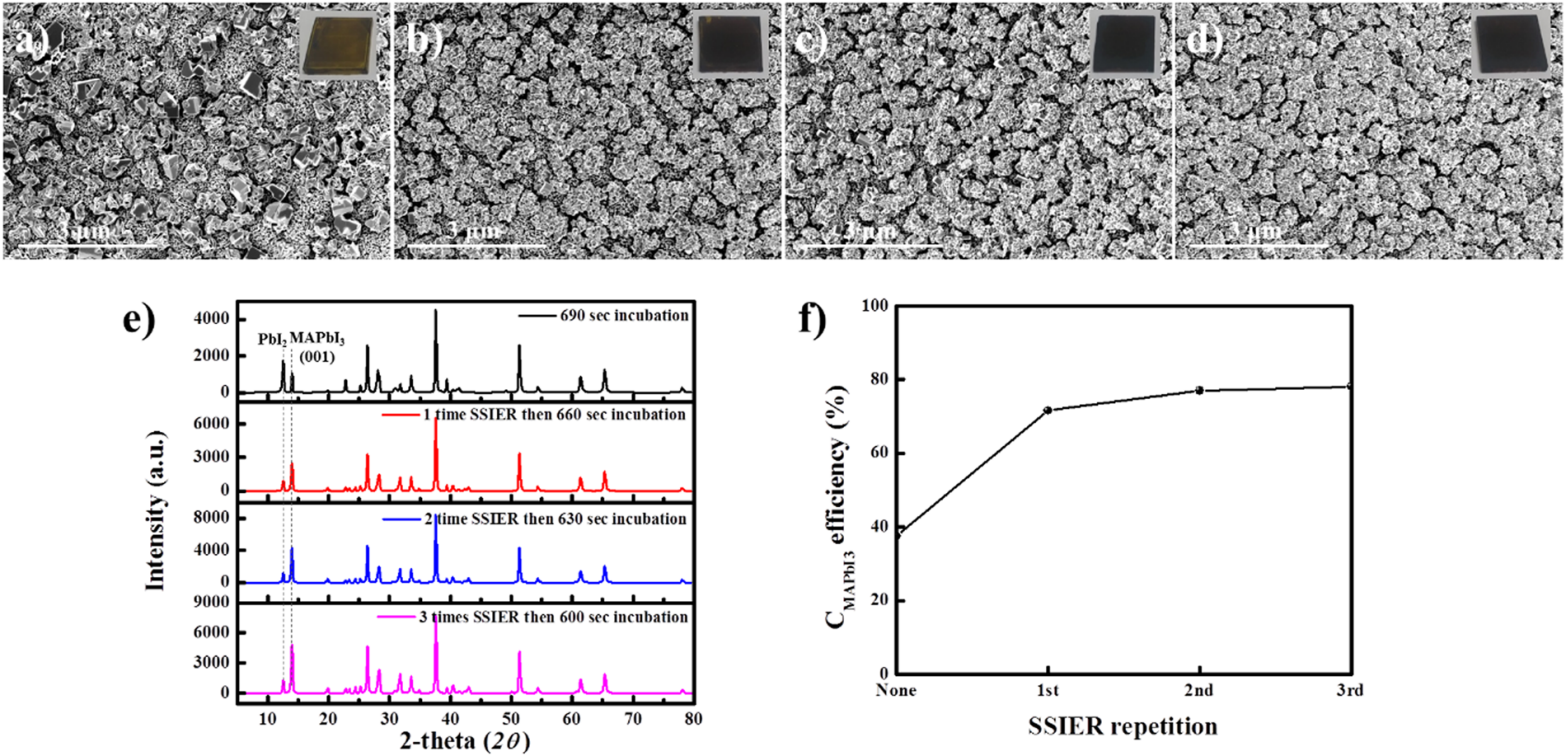
(**a**–**d**) The SEM surface morphologies with the corresponding pictures (inset) and (**e**) XDR patterns of the MAPbI_3_ perovskite layers fabricated by SSIER repetitions for a total MAI exposure time of 690 s. (**f**) The correlation between the perovskite conversion and SSIER repetition time.

A specific study regarding the SSIER was successfully performed as shown in Fig. 5. Figure 5a shows the SEM surface morphologies with their corresponding pictures (inset) and Fig. 5b shows XDR patterns for the MAPbI_3_ perovskite layers fabricated by SSIER repetitions for a total MAI exposure time of 690 s. The correlation between the perovskite conversion and SSIER repetition time is shown in Fig. 5c. These films were annealed at 80 °C for 10 min to completely remove the solvent. We used a Pb(NO_3_)_2_ layer deposited by dip-coating in an aqueous Pb(NO_3_)_2_ solution for a 30 s followed by drying at 120 °C.

As shown in Fig. 5a, even though these Pb(NO_3_)_2_ films were exposed for 690 s to the MAI solution with varied SSIER repetitions and long dipping times, the morphologies of the formed MAPbI_3_ film was significantly affected by the number of SSIER cycles. The decomposition of the MAPbI_3_ films was partially observed after the first cycle, as well as the films fabricated with no SSIER cycles. The MAPbI_3_ films formed after 2 SSIER cycles exhibited better stability (inset of Fig. 5a) compared to the 0 and 1 cycle films. Moreover, with more SSIER cycles, the surface coverage and crystallinity of the MAPbI_3_ perovskite films improved, as shown by the SEM surface morphologies and XRD patterns (Fig. 5a and b). Also, based on the XRD patterns, the conversion of MAPbI_3_ (*C*_MAPbI3_) could be qualitatively defined using the peak intensities of PbI_2_ and MAPbI_3_ at 2*θ* = 12.7° and 14.2°, respectively^7,31,32^.

1 $${C}_{{\rm{MAPbI}}3}={I}_{{12.7}^{^\circ }}/({I}_{{12.7}^{^\circ }}+{I}_{{14.2}^{^\circ }})$$

As shown in Fig. 5c, a greater number of SSIER repetitions gave higher conversion values for MAPbI_3_ perovskite generated from PbI_2_. These parameters are closely related by to the PbI_2_ derived from the ion-exchange reaction with the MAPbI_3_ perovskite and the unreacted Pb(NO_3_)_2_ in the solid state. It should be noted that the SSIER approach can provide superior stability as well as a better surface coverage and crystallinity of MAPbI_3_ perovskite films.

Although the SSIER repetition showed a well-developed surface coverage, crystallinity, and stability, it resulted in rather sparse surface coverage on the ZnO/m-TiO_2_/c-TiO_2_/FTO substrates, as shown in Fig. 5a. Inadequate surface coverage can lead to interfacial recombination between metal oxides and hole transporting materials (HTMs), resulting in the poor performance of the PrSC devices. Thus, a more complete surface coverage with the MAPbI_3_ perovskite films was attempted by introducing the SSD process in addition to the SSIER approach. Since the SSD process has been used for the step-by-step growth of nanoscale quantum dot layers^9^, we expected that the MAPbI_3_ perovskite deposited by SSD repetition could effectively improve the sparse coverage. In this study, the Pb(NO_3_)_2_ layer was deposited by dip-coating in an aqueous solution for 30 s, followed by drying at 120 °C. Then, it was exposed to the MAI solution for a 30 s to produce the MAPbI_3_ perovskite, followed by annealing at 80 °C. The above procedure describes a single SSD cycle.

**Figure 6:**
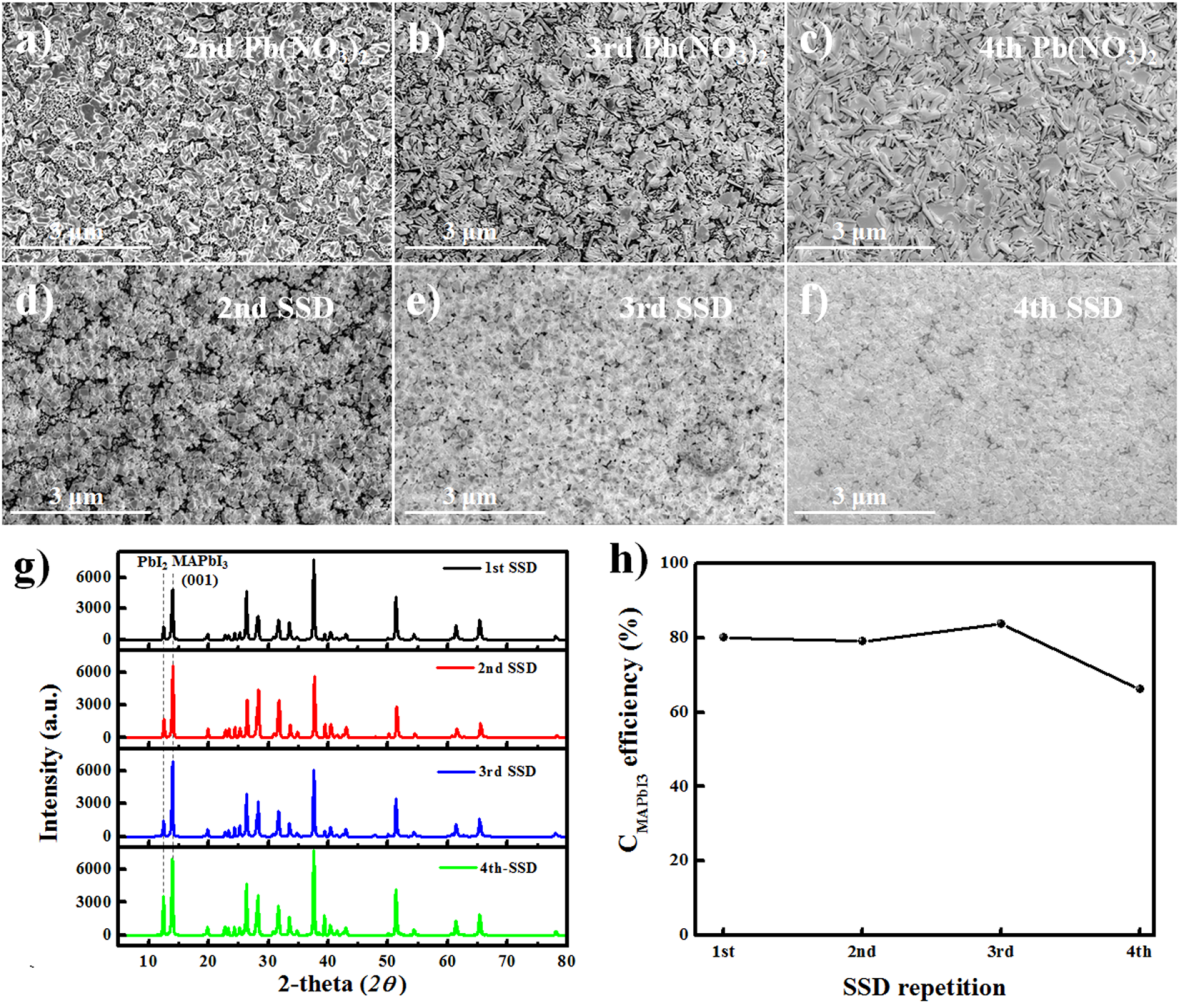
(**a**–**f**) The SEM surface morphologies of the MAPbI_3_ perovskite layers fabricated using the conventional SSD process through (**a**–**c**) ionic layer adsorption of Pb(NO_3_)_2_ from aqueous solution followed by (**d**–**f**) the reaction in MAI solution. (**g**) The XDR patterns of the MAPbI_3_ perovskite layers and (**h**) the correlation between the perovskite conversion and SSD repetition time.

Meanwhile, the surface morphologies of the MAPbI_3_ perovskite layers made by conventional SSD processes by (a–c) ionic layer adsorption of Pb(NO_3_)_2_ from aqueous solution followed by (d–f) the reaction in MAI solution, shown in Fig. 6. The XRD patterns of the MAPbI_3_ perovskite layers and the correlation between perovskite conversion and SSD repetition time are also shown in Fig. 6g.

As shown in Fig. 6a–c, when the as-formed MAPbI_3_ electrodes were exposed to aqueous Pb(NO_3_)_2_ solution, the water dissolved and removed the MAI from the MAPbI_3_ perovskite structure, leaving PbI_2_. A new layer of Pb(NO_3_)_2_ was simultaneously adsorbed on the surface of the ZnO/m-TiO_2_/c-TiO_2_/FTO substrate. Interestingly, more platelet structures of PbI_2_ were observed with increasing numbers of SSD cycles. In addition, a small increase in adsorption layer thickness was observed with the addition of Pb(NO_3_)_2_, even when comparing films after many SSD repetitions to those with only the first adsorption layer. This indicates that the additional Pb(NO_3_)_2_ is mainly adsorbed on the exposed ZnO/m-TiO_2_/c-TiO_2_/FTO because of the hydrophobicity of the PbI_2_ layers formed during the MAI removal in aqueous solution.

The MAPbI_3_ films were prepared via 3 SSIER cycles followed by a final incubation for 600 s in the MAI solution after each cycle of Pb(NO_3_)_2_ ionic layer adsorption (Fig. S4). As shown in Fig. 6(d–f), higher numbers of SSD repetitions results in superior surface coverage of the MAPbI_3_ perovskite films on the substrate. After the 3rd SSD cycle, there were fewer pin-holes in the MAPbI_3_ perovskite films compared to those fabricated with two or fewer cycles. It should be noted that the MAPbI_3_ perovskite deposited by SSD repetition can effectively improve the sparse coverage on the ZnO/m-TiO_2_/c-TiO_2_/FTO. Regarding the XRD patterns, the conversion of MAPbI_3_ (*C*_MAPbI3_) is independent of the 1st to 3rd SSD processes, but significantly decreased after the 4th SSD repetition, as shown in Fig. 6h. These results indicate that the crystallinity of the MAPbI_3_ perovskite films is mainly affected by the SSIER process, not the SSD process. Furthermore, SSIER might not be effective for perovskite crystal growth after the 4th SSD repetition.

### Photovoltaic performance of device fabricated with all sequential dip-coating processed perovskite film

**Figure 7:**
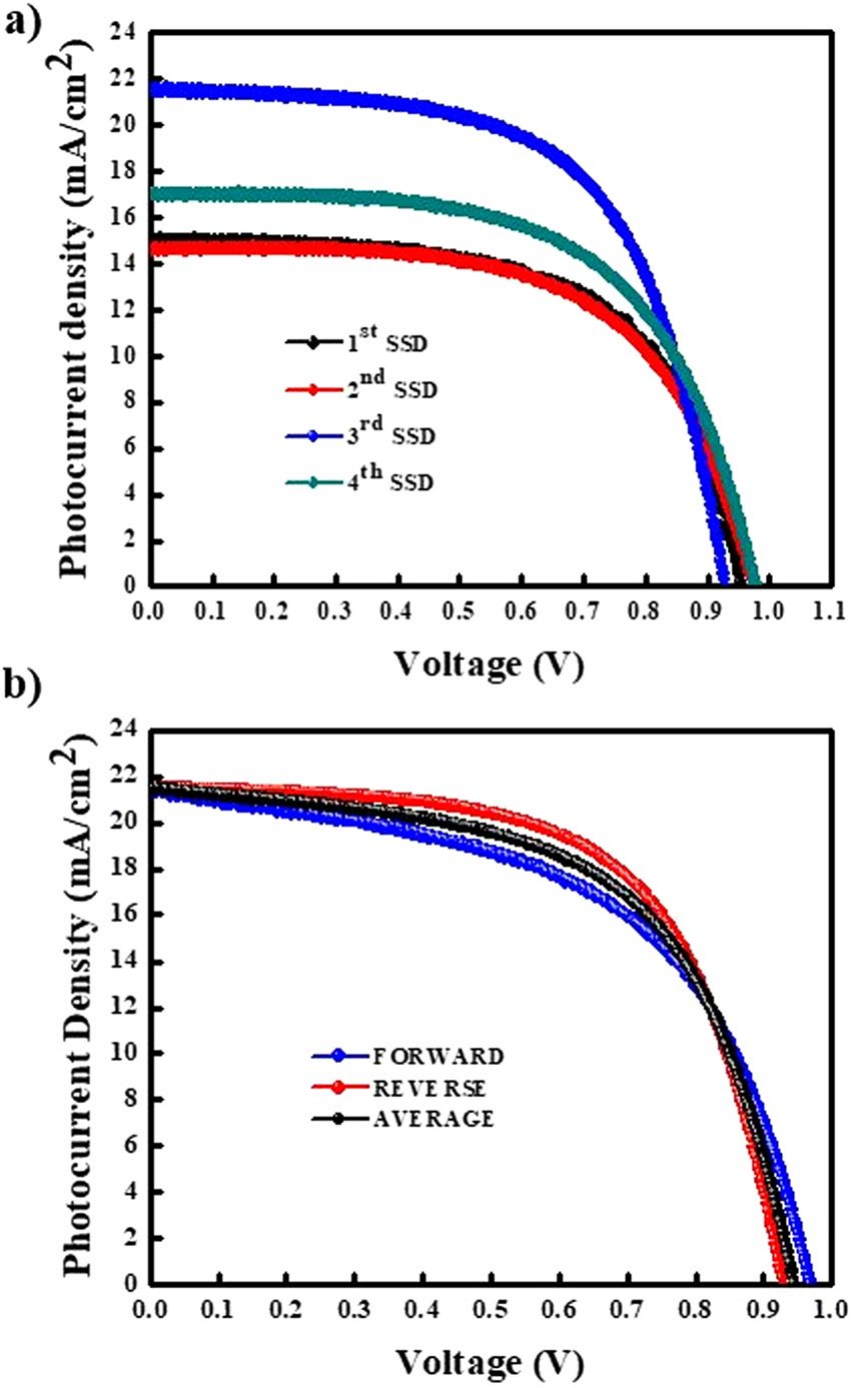
(**a**) Current-voltage (*J*-*V*) curves under AM 1.5 irradiation (100 mW·cm^−1^) for PrSCs based on MAPbI_3_ perovskite layers fabricated by a designated number of SSD repetitions followed by 3 SSIER cycles, with a final incubation for 600 s in the MAI solution. (**b**) The hysteresis behavior of the PrSCs fabricated under the optimized conditions (3 SSD and 3 SSIER processes, 600 s MAI incubation).

Figure 7a shows the current–voltage (*J*–*V*) curves under AM 1.5 irradiation (100 mW·cm^−1^) for PrSCs based on MAPbI_3_ perovskite layers fabricated by a designated number of SSD repetitions followed by three SSIER cycles, with a final incubation for 600 s in the MAI solution. Additionally, the hysteresis behaviors of PrSCs fabricated under the optimized conditions (3 SSD and 3 SSIER cycles, 600 s MAI incubation) are shown in Fig. 7b. The PrSCs were fabricated with an n-i-p structure of FTO/c-TiO_2_/m-TiO_2_/ZnO/MAPbI_3_/HTM/MoO_3_/Ag. The conductivity of 2,2′,7,7′-tetrakis(*N*,*N*-di-4-methoxyphenylamino)-9,9′-spirobifluorene (spiro-MeOTAD) and the efficiency of the HTM was improved with doping additives such as 4-tert-butylpyridine (t-BPy) and lithium bis(trifluoromethanesulfonyl) imide (Li-TFSI).

The optimization of the device performance is shown in Figs S5–9. As shown in Fig. 7a, the optimized PrSC devices exhibited the most efficient PCE (maximum/average) of 12.41/11.58% with a short-circuit current density (*J*_sc_) of 21.53 mA/cm^−2^, an open-circuit voltage (*V*_oc_) of 0.93 V, and a fill factor (*FF*) of 0.62. The PCEs of the devices based on MAPbI_3_ perovskite layers fabricated with different numbers of SSD cycles were not as high. One SSD cycle gave PCEs (maximum/average) of 8.73/7.01% with *J*_sc_ = 14.66 mA·cm^−2^, *V*_oc_ = 0.98 V, and *FF* = 0.61; two cycles gave PCEs of 9.07/8.05% with *J*_sc_ = 15.00 mA·cm^−2^, *V*_oc_ = 0.96 V, and *FF* = 0.63; and four cycles gave PCEs of 10.16/8.05% with *J*_sc_ = 17.00 mA·cm^−2^, *V*_oc_ = 0.98 V, and *FF* = 0.61. These results can be explained by the improved surface coverage and crystallinity of the films formed from sequential deposition with 3 SSD and 3 SSIER cycles, as shown in Fig. 4. In addition, the optimized PrSCs exhibited much higher performances than those based on non-halide precursors such as Pb(OAc)_2_ and Pb(ClO_4_)_2_ (Fig. S10).

Meanwhile, the typical n-i-p PrSCs fabricated with the m-TiO_2_ electrode often show hysteretic *J*-*V* behavior depending on the scan direction (reverse or forward) due to differing charge extraction or transportation rates of holes and electrons separated from excitons^10^. Fig. 7b shows the hysteresis of the *J*-*V* curves in both scan directions under AM 1.5 irradiation (100 mW·cm^−1^) for the optimized PrSC. The hysteresis behaviors of the PrSCs are shown in Fig. 7b and are summarized in Fig. S11 and Table S1. Most of PrSCs exhibited negligible differences in *J*_sc_ and *V*_oc_ values in both directions, but the *FF* values were significantly reduced in the forward direction. Nonetheless, the average of the PCE values obtained in both directions was approximately 6% lower than the PCE value in the reverse direction, showing good external quantum efficiency (EQEs) in the light absorption region (Fig. S12).

### Large surface area perovskite films

**Figure 8:**
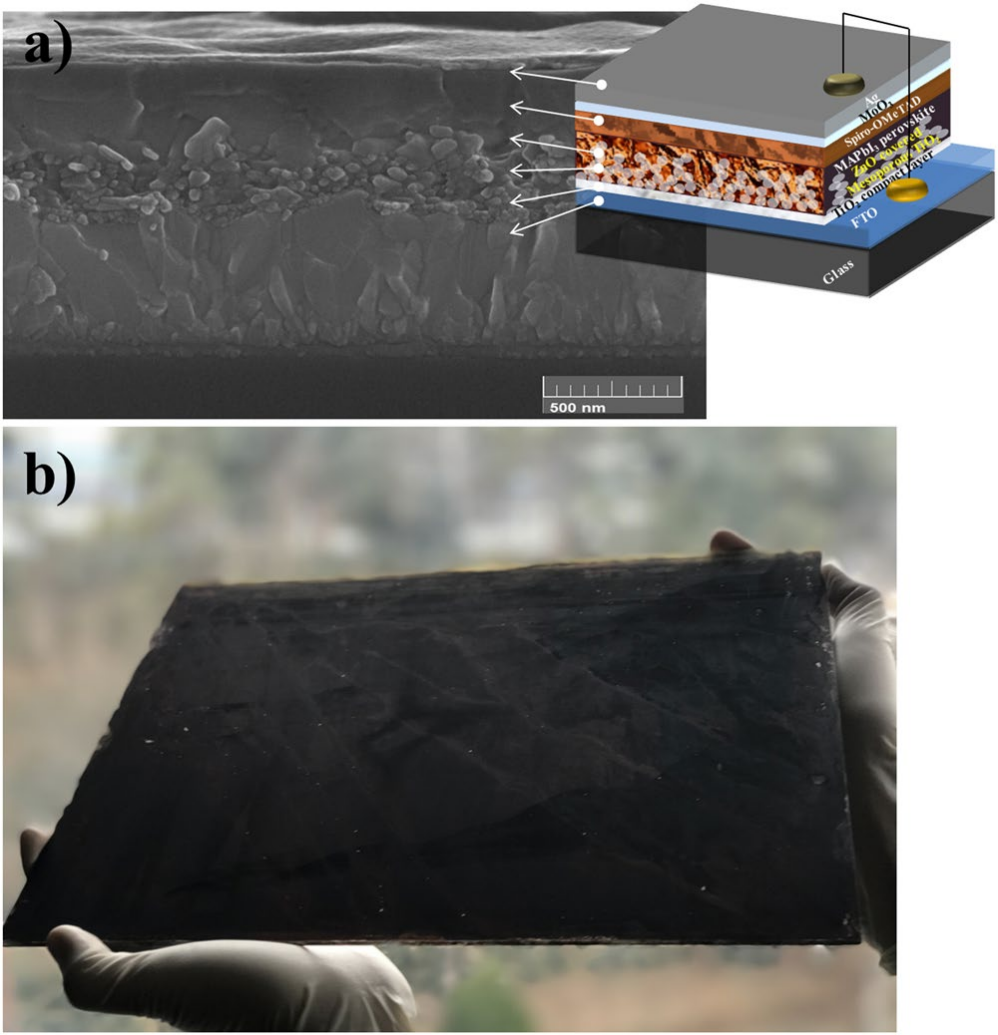
(**a**) The SEM image of a cross-section of the PrSC device fabricated using 3 SSDs and 3 SSIER repetitions with a final incubation of 600 s in the MAI solution. (**b**) Photograph of a large area MAPbI_3_ perovskite film fabricated by sequential SSD and SSIER deposition on large area glass substrate of 780 cm^2^ (30 cm (width) × 26 cm (height)).

Next, we characterized the cross-sectional morphology of the optimized PrSC device. Figure 8a shows the SEM image of a cross-section of the PrSC device fabricated using 3 SSD and 3 SSIER cycles with a final incubation for 600 s in the MAI solution. A ∼100 nm thin section was prepared using the focused ion beam (FIB) technique to investigate the vertically formed morphologies and interfacial heterojunctions between the MAPbI3 perovskite, ZnO, and m-TiO_2_/c-TiO_2_ electrodes.

As shown in Fig. 8a, the MAPbI_3_ perovskite layer was well organized between the spiro-MeOTAD HTM and ZnO/m-TiO_2_/c-TiO_2_/FTO substrate without interfacial dead spaces. However, the film was non-uniform and thin with a thickness of approximately 250 nm, in contrast to the high efficiency PrSCs reported previously. Herein we prepared a large area perovskite film using a sequential dip-coating deposition approach with a non-halide lead precursor in aqueous solution to facilitate a green and low-cost manufacturing process. The photograph in Fig. 8b shows the MAPbI_3_ perovskite films fabricated by a sequential SSD and SSIER deposition approach on large area glass substrates. To demonstrate a large area MAPbI_3_ perovskite film, we prepared a thin ZnO layer on a bare FTO substrate of 780 cm^2^ (30 cm × 26 cm, width × height) using the dip-coating method. Then, the MAPbI_3_ perovskite films were deposited with the sequential SSD and SSIER approach. Even though this large area MAPbI_3_ perovskite film was non-uniform due to the uneven surface morphology of the ZnO under layer, we successfully realized a large area perovskite film using the sequential dip-coating deposition approach with a non-halide lead precursor in an aqueous solution. This represents significant progress towards environmentally benign and low-cost manufacturing processes for large area PrSCs.

## Conclusion

We have demonstrated an efficient approach for preparing large area MAPbI_3_ perovskite films using sequential dip-coating deposition with ionic layer adsorption of Pb(NO_3_)_2_ in aqueous solvent followed by reaction with MAI. This represents significant progress toward an environmentally benign and low-cost manufacturing processes for large area PrSCs. Notably, the ZnO interfacial layer induced significant adsorption of Pb(NO_3_)_2_ from aqueous solution within a very short period. A new SSIER approach was developed to prevent the decomposition of MAPbI_3_ perovskite structures formed from Pb(NO_3_)_2_ and MAI, which was rapidly followed by ion-exchange reactions with unreacted Pb(NO_3_)_2_ even in the solid-state. The method developed herein afforded superior stability, surface coverage, and crystallinity of MAPbI3 perovskite films compared to those prepared without the SSIER process. In addition, the introduction of the SSD process to the SSIER approach led to more complete surface coverage with the MAPbI_3_ perovskite films affording superior morphology, stability, and crystallinity. The PrSCs based on these sequential depositions of the MAPbI_3_ perovskite layer using aqueous Pb(NO_3_)_2_ solution exhibited excellent PCEs of 12.41%, which is comparable to films fabricated from spin-casting of halide or non-halide lead precursors. The results of this study lay the groundwork for the development of environmentally benign and low-cost manufacturing of high efficiency and large area PrSCs.

## Methods

### Materials

The MAPbI_3_ was prepared according to a method reported previously^1^. All solvents were purchased from Sigma-Aldrich, TCI, and Alfa Aesar and were purified using appropriate methods. The MAPbI_3_ precursor solution was prepared under a nitrogen atmosphere. Spiro-OMeTAD was obtained from Solaronix.

### Fabrication of MAPbI_3_ perovskite films

For the fabrication of MAPbI_3_ perovskite film, we applied a novel SSIER approach. A metal oxide-coated FTO substrates was first dipped into a 0.1 M solution of Pb(NO_3_)_2_ (Sigma-Aldrich, 99.9%) dissolved in ethanol/water (2:1, v/v) for approximately 30 s. The substrate was washed with deionized (DI) water and ethanol and then annealed at 120 °C for 10 min, resulting in the formation of a transparent film. The substrate was dipped into a 0.1 M MAI solution in isopropanol (Sigma-Aldrich, 99.5%), for 30 s, washed with chloroform and diethyl ether (Sigma-Aldrich, >96%), and annealed at 80 °C for 10 min. The above procedure represents a single SSIER cycle. After the desired number of SSIER repetitions, the prepared films were exposed for 600 s to the MAI solution, followed by annealing at 80 °C for 10 min. In addition to the SSIER process, the SSD process was performed. In the first cycle of the SSD process, the metal oxide-coated FTO substrates were dipped into a 0.1 M solution of Pb(NO_3_)_2_ dissolved in ethanol/water (2:1, v/v) for approximately 30 s, washed using pure water and ethanol, and annealed at 120 °C for 10 min. The Pb(NO_3_)_2_ layer-coated substrate was then dipped into the MAI solution for 30 s followed by annealing at 80 °C for 10 min. The above procedure represents a single SSD cycle. After the designated number of SSD repetitions, the SSIER process was subsequently performed to provide an improved MAPbI_3_ perovskite film.

### PrSC device fabrication

A clean FTO was UVO treated for 10 min. The TiO_2_ blocking layer (bl-TiO_2_) was spin coated on a FTO glass substrate with a titanium(IV) diisopropoxide bis(acetylacetonate) solution diluted in butanol (1:10, v/v) at 700 rpm for 8 sec, 1000 rpm for 10 sec and 2000 rpm for 40 sec followed by dryig at 125 °C for 5 min. Mesoporous titanium oxide (mp-TiO_2_) layer was spin coated on the bl-TiO_2_ with 1.2 g of TiO_2_ nanoparticles (40 nm size from ENB Korea) diluted in 10 ml of anhydrous ethanol solution at a speed of 2000 rpm for 20 sec and finally annealed at 550 °C for 1 hr and the UVO treated for 15 min. The ZnO sol-gel was synthesized by reacting zinc acetate dihydrate (1.6 g, Sigma Aldrich), ethanolamine (0.5 g, Sigma Aldrich) and 2-methoxy-ethanol (10 ml, Sigma Aldrich) and stirr for 4 hrs at room temperature and spin coated over the mp-TiO_2_ at 5000 rpm for 40 sec and finally annealed at 300 °C for 1 hr^33^. MAPbI_3_ layers were fabricated according to the method described above. The Spiro-OMeTAD HTM was deposited via spin-coating at 3000 rpm for 30 s. The solution was prepared by mixing 29 mg of Spiro-OMeTAD, 7 μL of 170 mg/ml Li-TFSI in acetonitrile, and 11 μL of 4-tBPy. Finally, device fabrication was completed by thermal evaporation of the thin MoO_3_ layer and thick Ag layer on top of the HTM film under reduced pressure (less than 10^−6^ Torr).

### Measurements and Instrument

The absorption spectra were recorded on a PerkinElmer Lambda 2S ultraviolet (UV)-visible spectrometer. The surface morphologies were investigated using a field emission scanning electron microscope (FESEM, Nova Nano-SEM 450, FEI, Netherlands). The perovskite crystallinities of the MAPbI3 layers were determined using X-ray diffraction (XRD, D/Max2500 V/PC, Rigaku Corp, Japan). The solar cell efficiencies were characterized under simulated 100 mW·cm^−1^ AM 1.5 G irradiation from a Xe arc lamp with an AM 1.5 global filter. The simulator irradiance was characterized using a calibrated spectrometer; the illumination intensity was set using a silicon diode with an integrated KG1 optical filter certified by the National Renewable Energy Laboratory (NREL). The spectral mismatch factors were less than 5% for each device. The short circuit currents were also within 5% of the values calculated using the integrated EQE spectra and the solar spectrum. The applied potential and cell currents were measured using a Keithley 2400 model digital source meter. The *J*-*V* curves were measured at a voltage settling time of 100 ms. The EQEs were measured by under-filling the device area using a reflective microscope objective to focus the light output from a 75 W Xe lamp, monochromator, and optical chopper. The photocurrent was measured using a lock-in amplifier and the absolute photon flux was determined using a calibrated silicon photodiode and was recorded for 5 s per point (80 points) from 350 to 900 nm. In the *J*-*V* curves hysteresis tests, the “forward” scan means to measure the sweeping voltage from the short circuit to forward bias, whereas a “backward” scan means to sweep in the opposite direction. To explore the active area of device and avoid scattering effects from the edges, a non-reflective metal plate mask with an aperture of 4.5 mm^2^ was used for the solar cells.

## Electronic supplementary material

Supplementary Information
