# Supplementary material for: All Sequential Dip-Coating Processed Perovskite Layers from an Aqueous Lead Precursor for High Efficiency Perovskite Solar Cells
Source: Sci Rep. 2018 Feb 1;8:2168. doi: 10.1038/s41598-018-20296-2 (PMC5794860; doi:10.1038/s41598-018-20296-2)
Supplement: Supplementary file 1 — Supplementary Information [file 41598_2018_20296_MOESM1_ESM.pdf]

Supplementary information

All Sequential Dip-Coating Processed Perovskite Layers from an Aqueous Lead Precursor for High Efficiency Perovskite Solar Cells

Muhammad Adnan[1] and Jae Kwan Lee[1,2]*

[1]*Department of Chemistry, Graduate School, Chosun University, Gwangju, 501-759, Republic of Korea.*

[2]*Department of Chemistry Education/Carbon Materials, Chosun University, Gwangju, 501-759, Republic of Korea.*

*E-mail: chemedujk@chosun.ac.kr

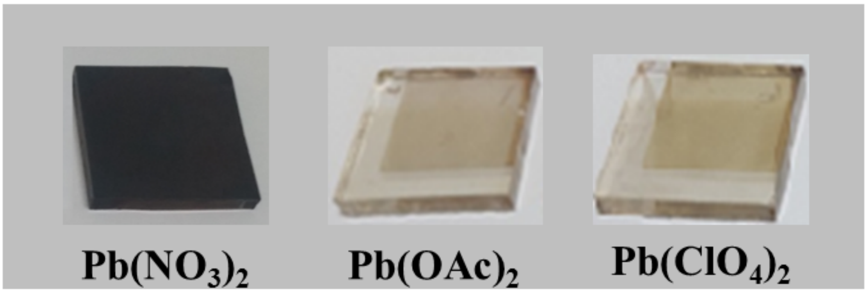

Figure S1. The photo images of $MAPbI_3$ perovskite layers obtained through ionic layer adsorption of $Pb(NO_3)_2$, $Pb(OAc)_2$, and $Pb(ClO_4)_2$ from aqueous solution for 30 sec and reaction for 600 sec in MAI solution

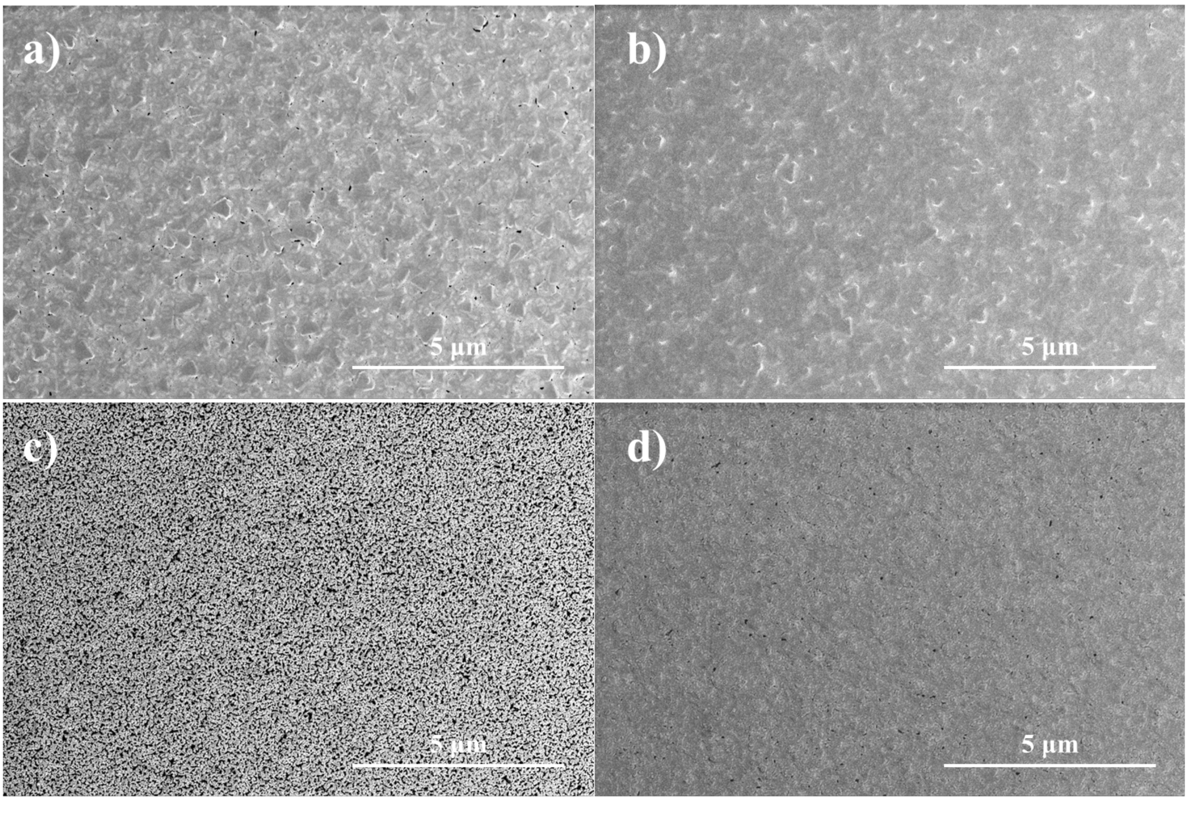

Figure S2. ZnO thin layer covered TiO_2 film prepared from spin-casting of sol-gel precursor solution followed by annealing at the temparature of 300°C on FTO substrate: (a) compact (c-) TiO_2, (b) ZnO/c- TiO_2, (c) mesoporous (m-) TiO_2 /c- TiO_2, (d) ZnO/m- TiO_2 /c-TiO_2

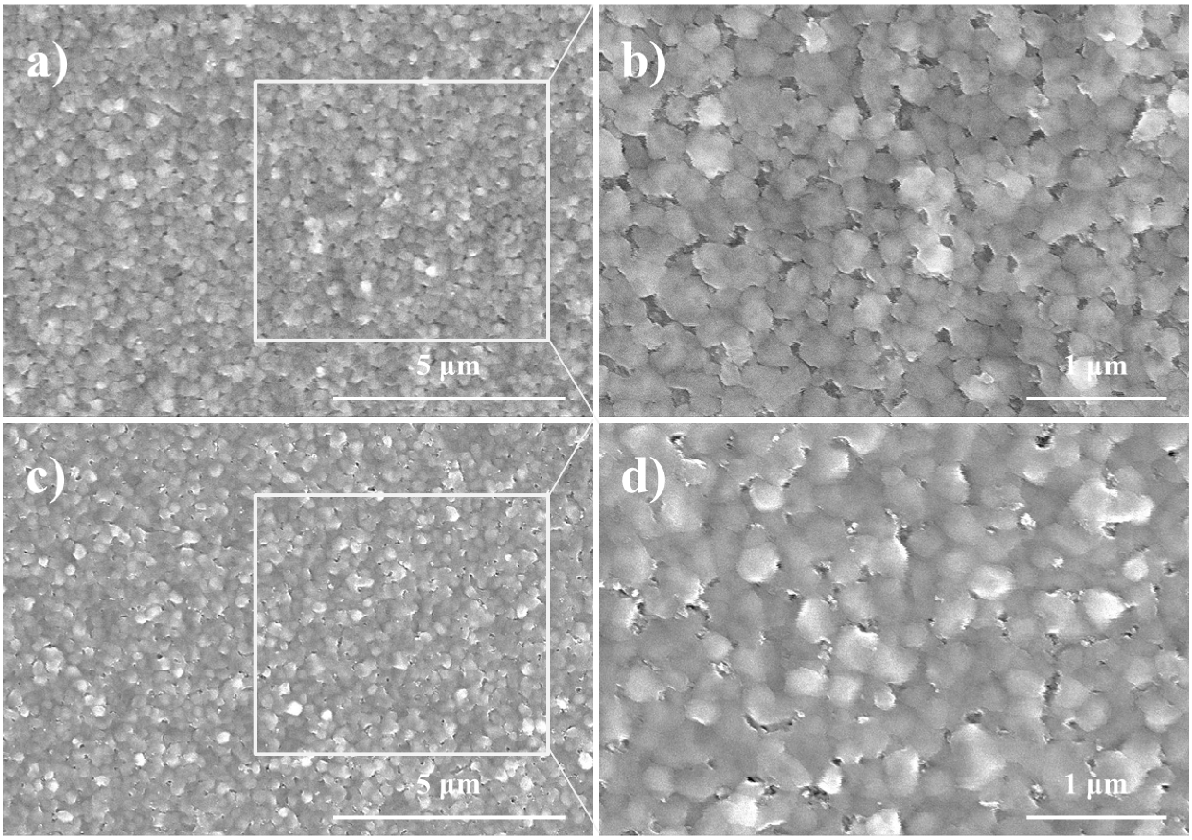

Figure S3. SEM surface morphologies for ionic layer adsorption of $Pb(NO_3)_2$ on m-TiO_2/c-TiO_2/FTO substrates formed when the substrates were dipped in $Pb(NO_3)_2$ solution (H_2O/ethanol, 1:1 v/v) for (a, b) 30 sec and (c, d) 10 min.

.

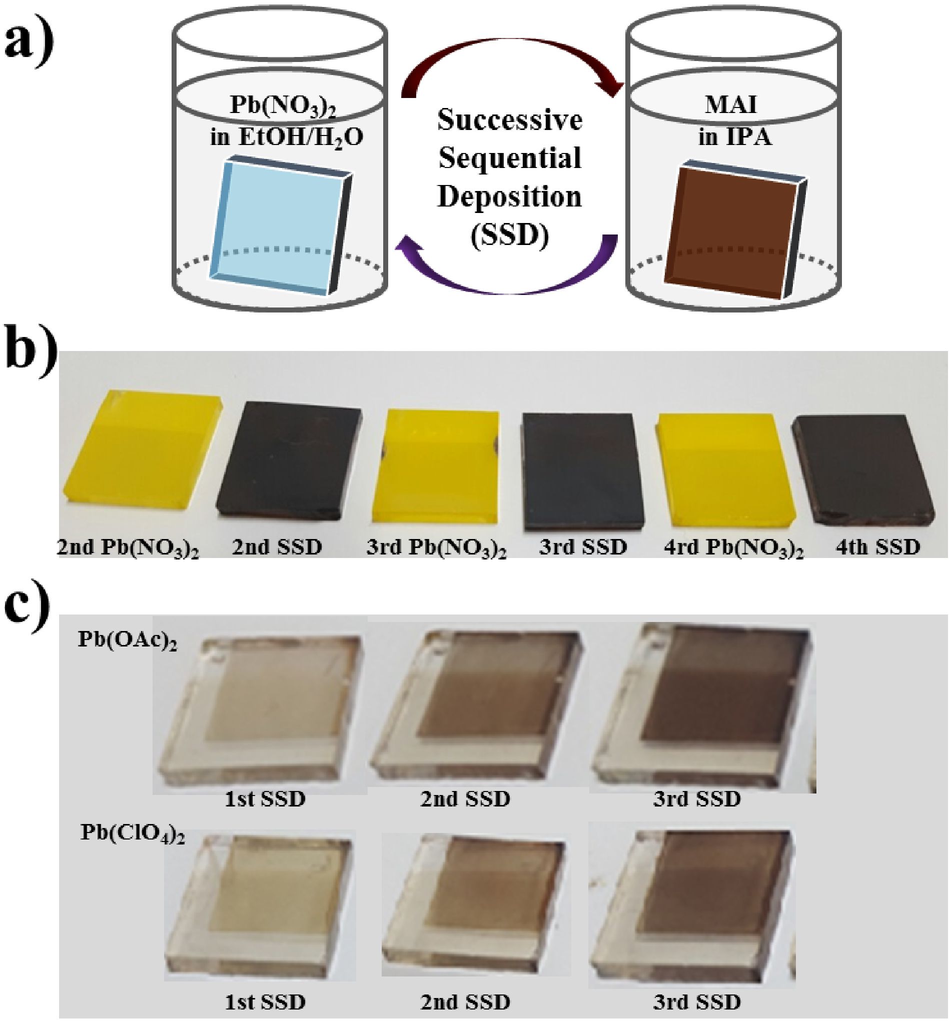

Figure S4. Schematic description using (a) the SSD and (b) the photo images PbI_2 and $MAPbI_3$ perovskite layers obtained through ionic layer adsorption of $Pb(NO_3)_2$ from aqueous solution and reaction in MAI solution, respectively. These were compared with those based on $Pb(OAc)_2$ and $Pb(ClO_4)_2$. All samples of $MAPbI_3$ perovskite layers after the designed SSD repetition were proceeded by 3rd SSIER with final incubation for 600 sec in MAI solution
.

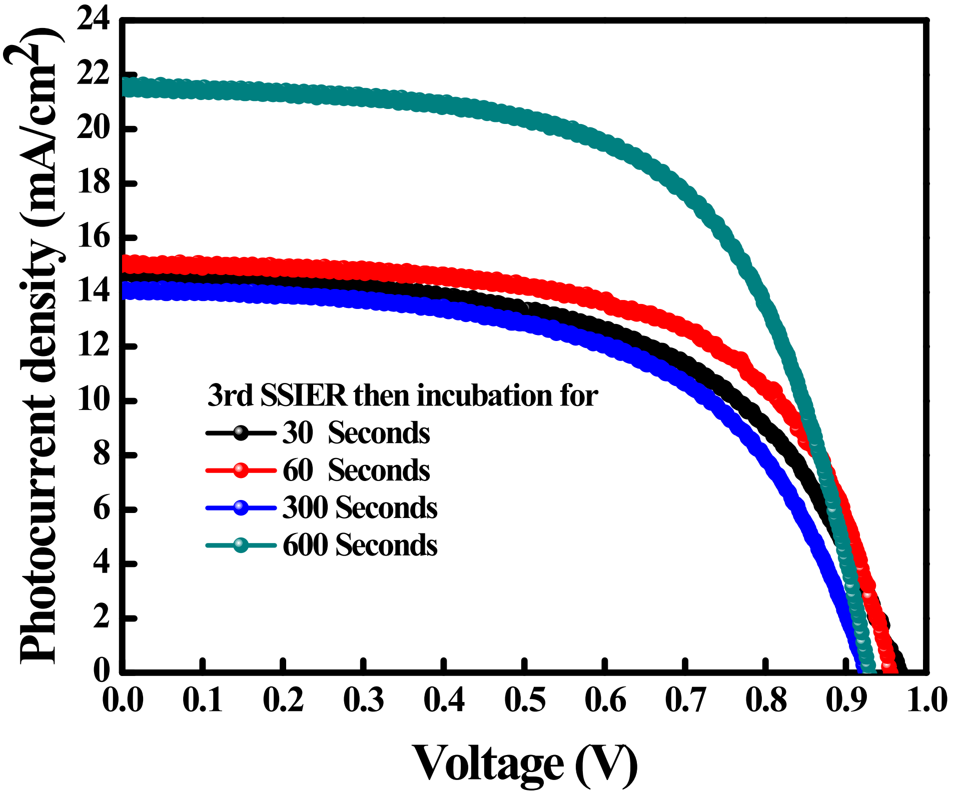

Figure S5. Device performances of PrSCs fabricated from $MAPbI_3$ perovskite layers with various final incubation times in MAI solution after a sequential deposition with the 3rd SSD followed by 3rd SSIER repetition.

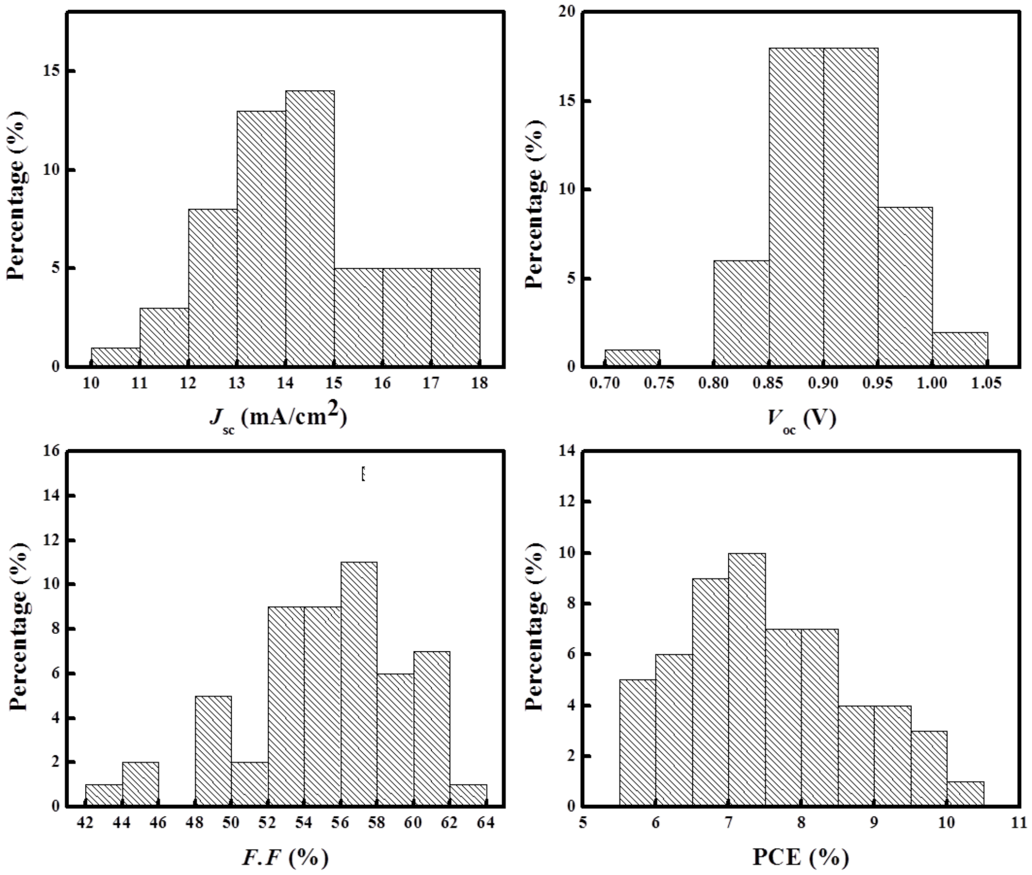

Figure S6. Device performances statistics based on more than 40 PrSCs fabricated by $MAPbI_3$ perovskite layers after the sequential deposition with the 1st SSD followed by 3rd SSIER repetition, then incubated for 600 sec in MAI solution

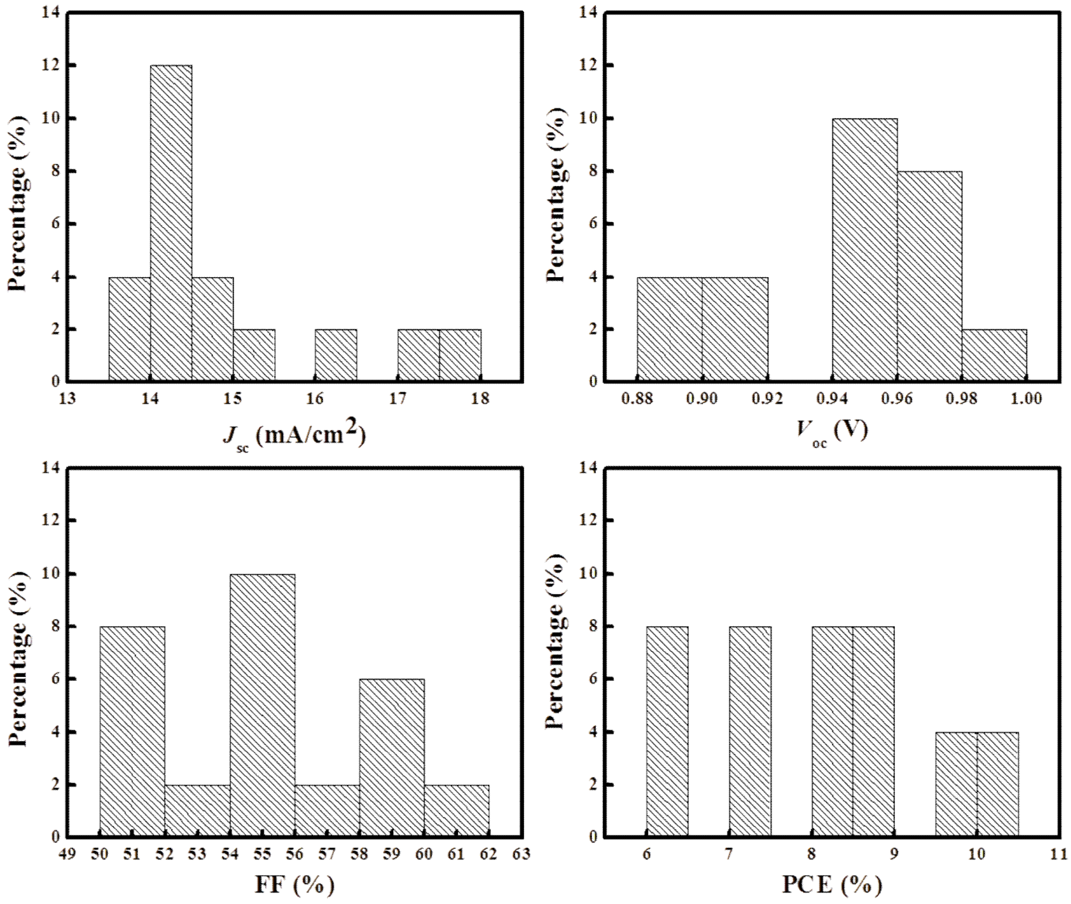

Figure S7. Device performances statistics based on more than 40 PrSCs fabricated by $MAPbI_3$ perovskite layers after the sequential deposition with the 2nd SSD followed by 3rd SSIER repetition, then incubated for 600 sec in MAI solution.

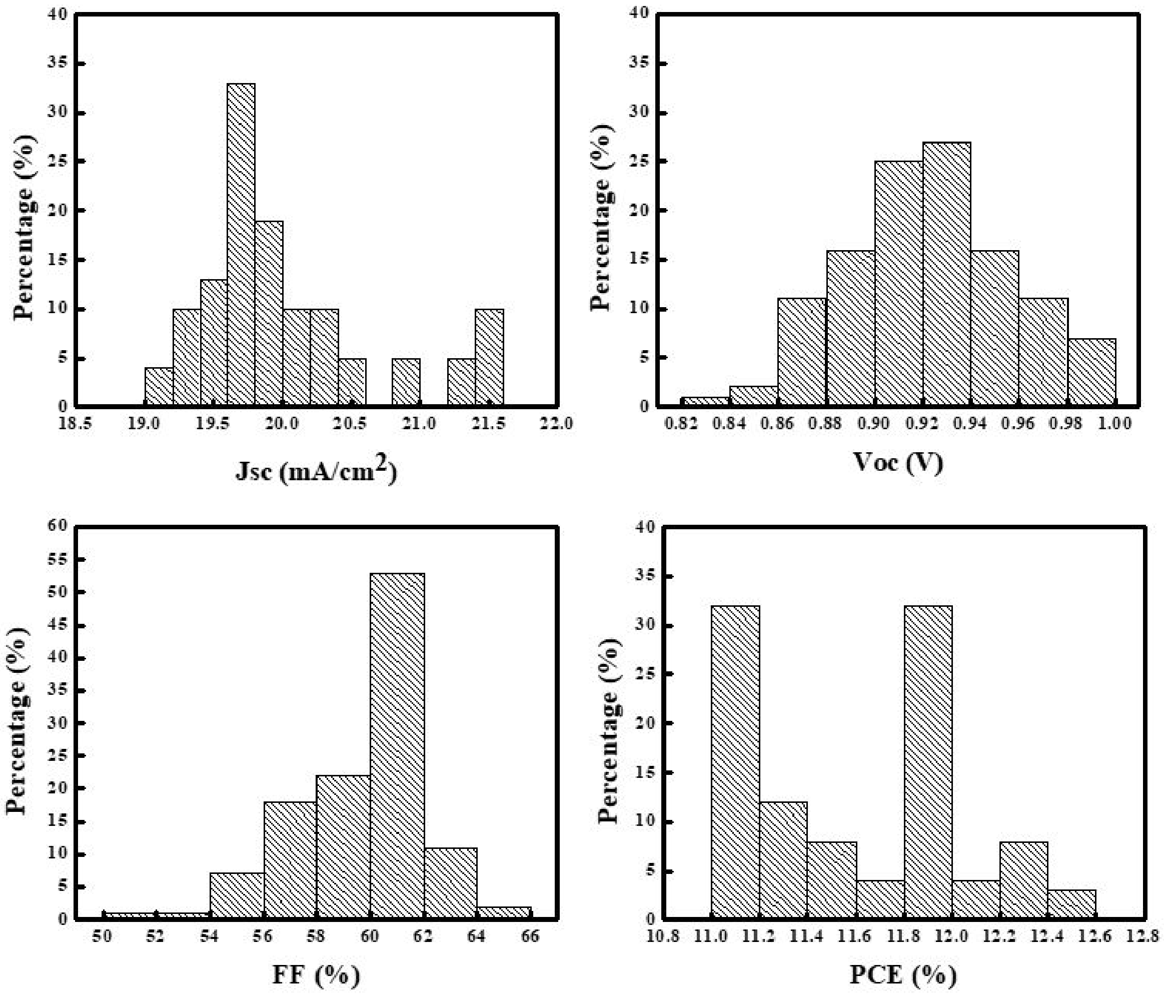

Figure S8. Device performances statistics based on more than 40 PrSCs fabricated by $MAPbI_3$ perovskite layers after the sequential deposition with the 3rd SSD followed by 3rd SSIER repetition, then incubated for 600 sec in MAI solution.

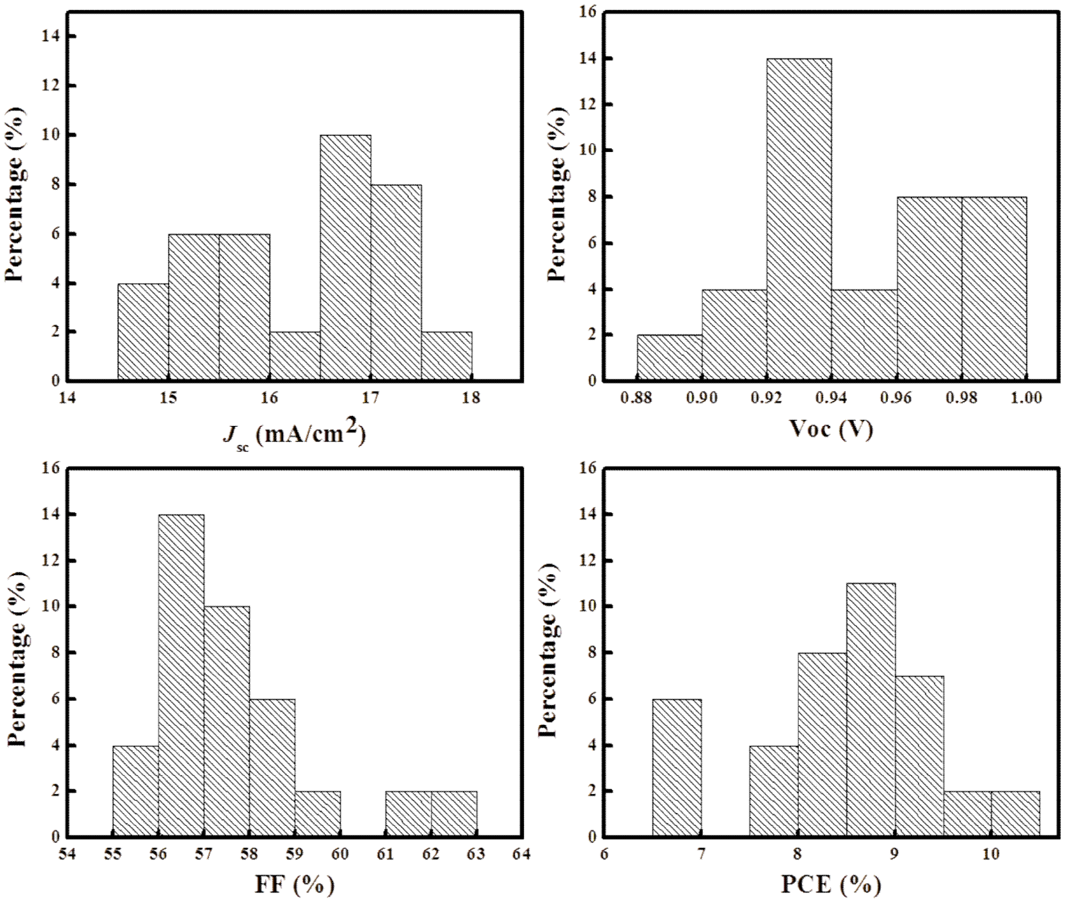

Figure S9. Device performances statistics based on more than 40 PrSCs fabricated by $MAPbI_3$ perovskite layers after the sequential deposition with the 4th SSD followed by 3rd SSIER repetition, then incubated for 600 sec in MAI solution.

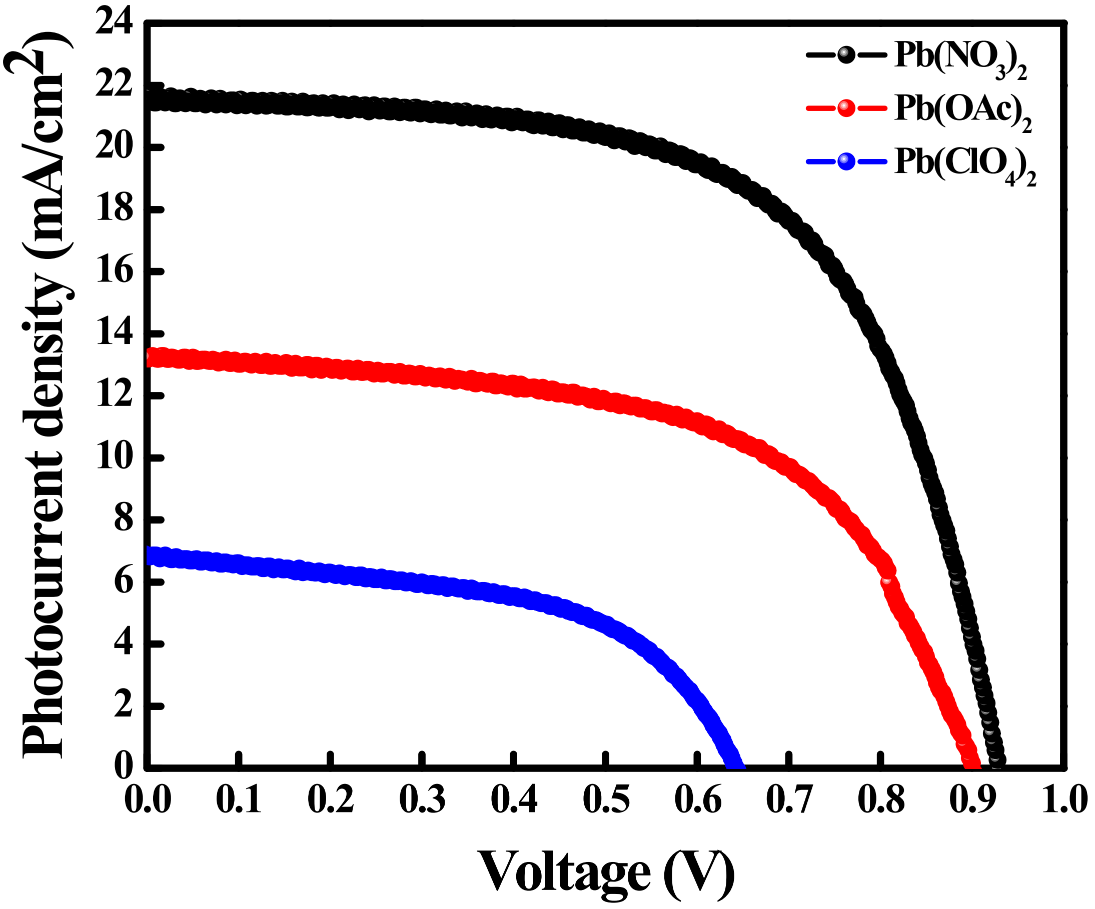

Figure S10. The optimized device performances of PrSCs fabricated with $MAPbI_3$ perovskite layers based on the $Pb(NO_3)_2$ (black), $Pb(OAc)_2$ (red), and $Pb(ClO_4)_2$ (blue) precursor using SSD and SSIER processes.

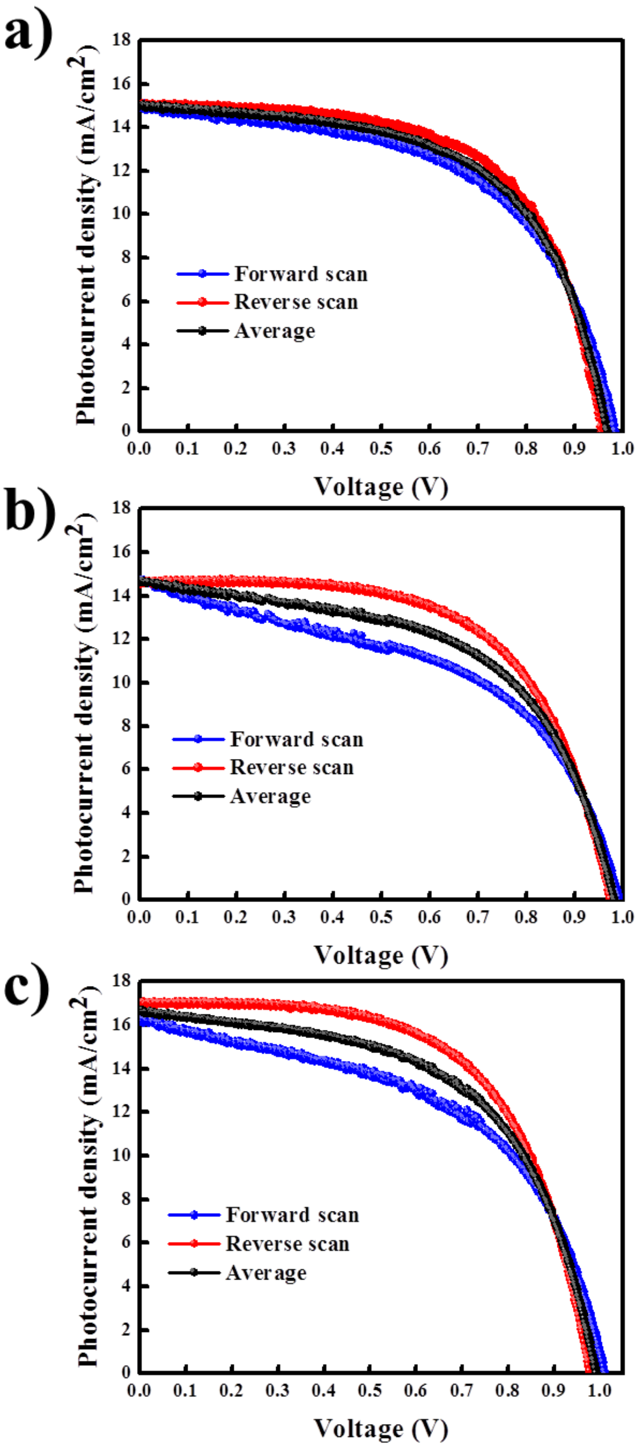

Figure S11. Hysteresis behaviors of PrSCs fabricated under the optimized condition ((a) 1st, (b) 2nd, and (c) 4th SSD and 3rd SSIER processes, 600 sec MAI incubation)

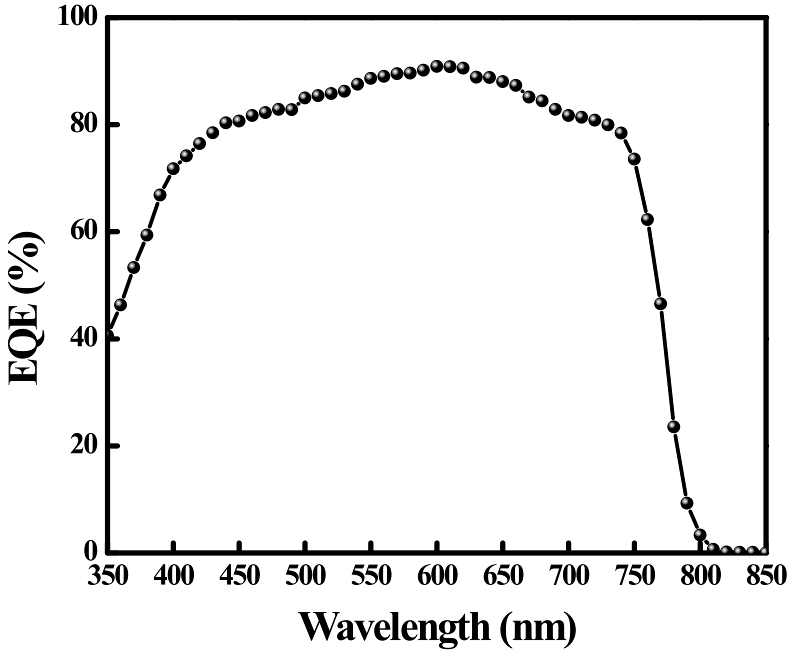

Figure S12. EQE spectra of PrSCs fabricated under the optimized condition with $MAPbI_3$ perovskite layers using a sequential deposition with the 3rd SSD followed by 3rd SSIER repetition.

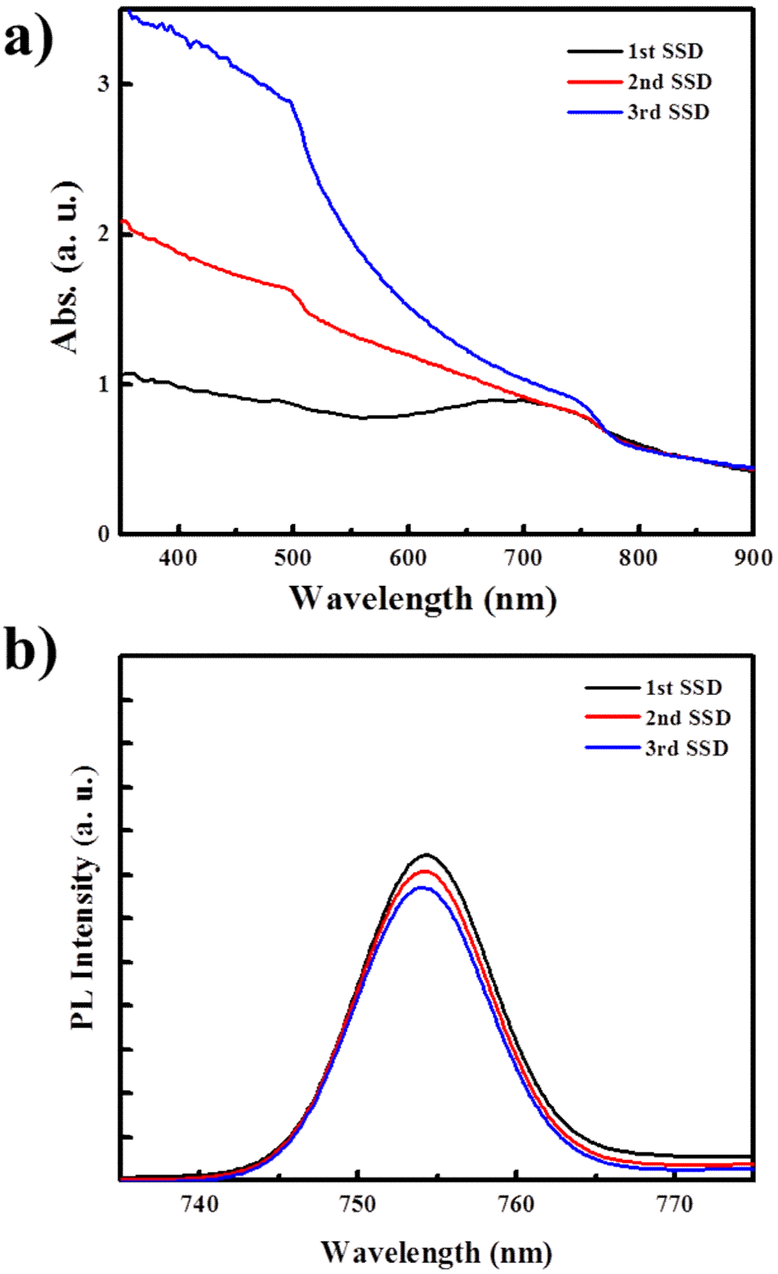

Figure S13: (a) UV-Visible spectra and (b) photoluminescene spectra of $MAPbI_3$ layers, which were fabricated based on 1st (black line), 2nd (red line), and 3rd SSD with 3rd SSIER process, on FTO/c-TiO_2/m-TiO_2/ZnO substrate.

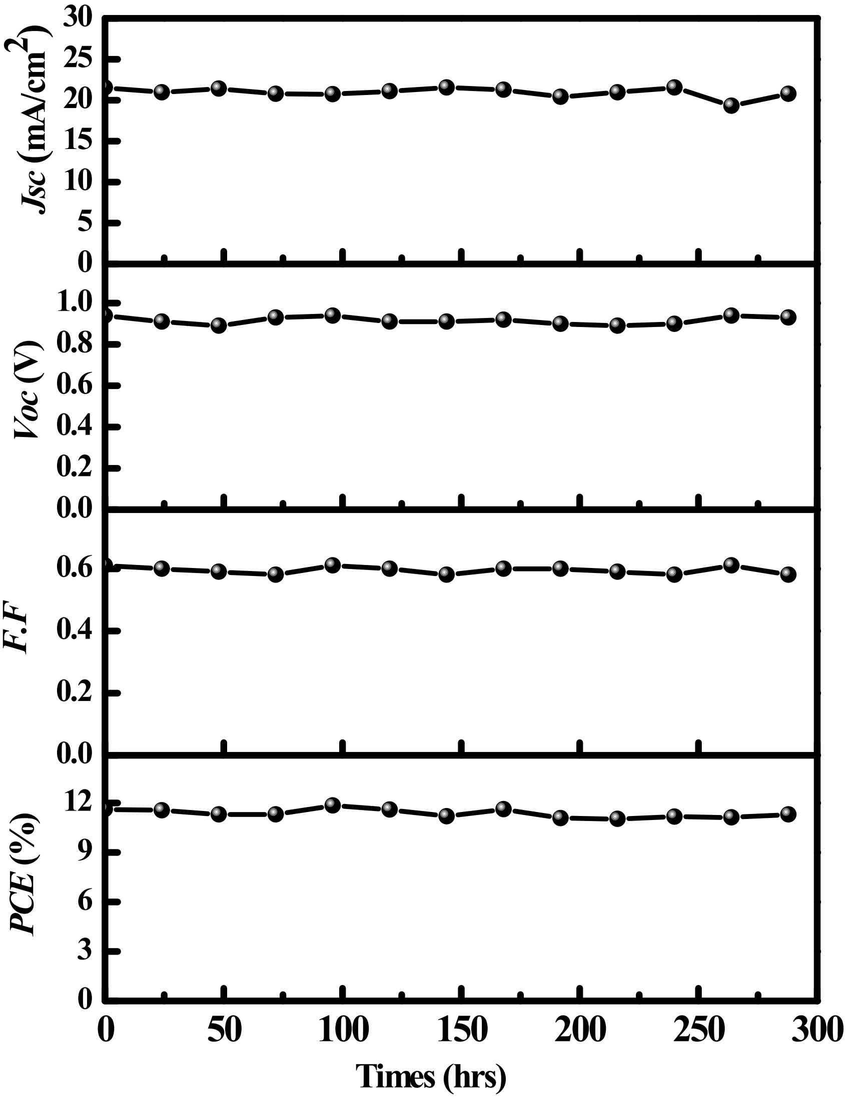

Figure S14: Photovoltaic performance of device, which was fabricated based on 3rd SSD with 3rd SSIER process, versus time. Stability tests were carried out keeping devices at a relative humidity of 10% without any encapsulation for 300 hrs.

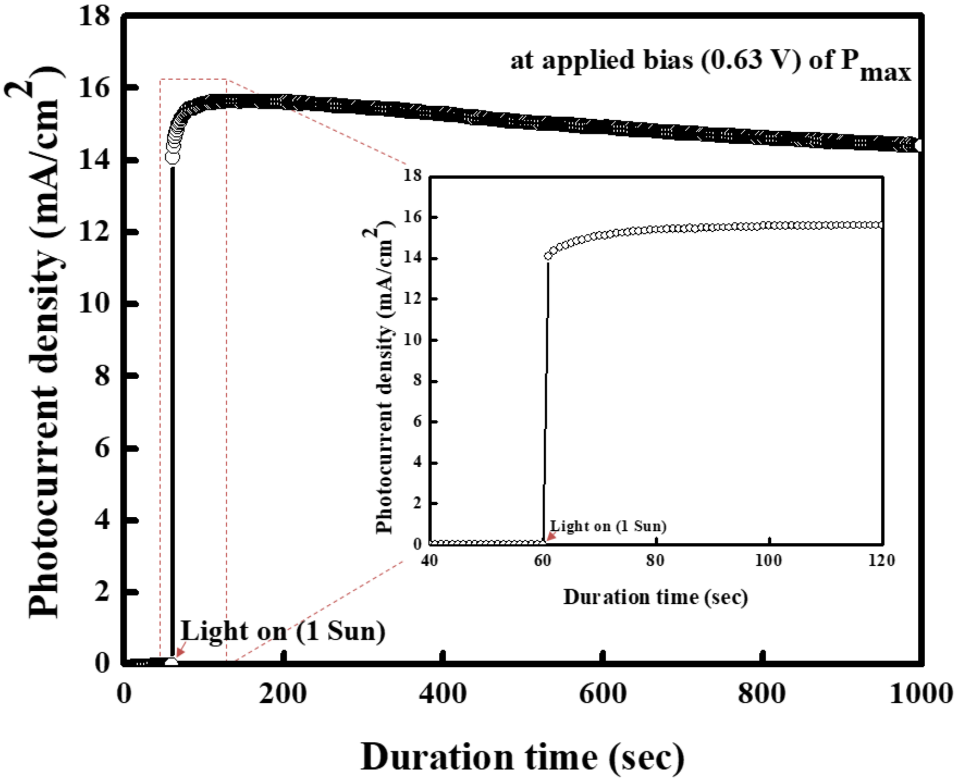

Figure S15: Photocurrent density as a function of time using 3rd SSD and 3rd SSIER with 600 sec MAI incubation time optimized device held at a bias of maximum 0.63 V output power point.

Table S1. Photovoltaic performances based on the hysteresis behaviors of PrSCs fabricated under the optimized condition.[a]

SSD cycles	Scan	J_{sc} (mA/cm^2)	V_{oc} (V)	*F·F*	η (%)
	Forward	14.71	0.99	0.49	7.14
**1st**	Reverse	14.60	0.98	0.61	8.73
	Average	14.66	0.99	0.55	7.94
	Forward	14.96	0.99	0.55	8.15
**2nd**	Reverse	15.00	0.96	0.63	9.07
	Average	14.98	0.98	0.59	8.61
	Forward	21.42	0.97	0.54	11.22
**3rd**	Reverse	21.53	0.93	0.62	12.41
	Average	21.47	0.95	0.58	11.81
	Forward	16.36	1.01	0.52	8.59
**4th**	Reverse	17.00	0.98	0.61	10.16
	Average	16.68	1.00	0.57	9.60

[a)] The performances are determined under simulated 100 mW·cm^{-2} AM 1.5 illumination. The light intensity using calibrated standard silicon solar cells with a proactive window made from KG5 filter glass traced to the NREL. The masked active area of device is 4.5 mm^2.
